# Scindapsus Aureus Resistive Random-Access Memory with Synaptic Plasticity and Sound Localization Function

**DOI:** 10.3390/nano15090659

**Published:** 2025-04-26

**Authors:** Lu Wang, Jiachu Xie, Wantao Su, Zhenjie Du, Mingzhu Zhang

**Affiliations:** School of Electronic Engineering, Heilongjiang University, Harbin 150080, China

**Keywords:** bio-memristor, synaptic plasticity, paired-pulse facilitation (PPF), spike-timing-dependent plasticity (STDP), interaural time difference (ITD), neuromorphic auditory system, green electronic materials

## Abstract

This work presents a memristive device based on a composite of Scindapsus aureus (SA) and gold nanoparticles (Au NPs), which exhibits excellent resistive switching characteristics and supports multiple forms of synaptic plasticity such as paired-pulse facilitation (PPF), spike-rate-dependent plasticity (SRDP), and spike-timing-dependent plasticity (STDP). The device demonstrates reliable retention, reproducibility, and switching stability. The SA:Au NP composite originates from a natural plant source and possesses green, biodegradable, and biocompatible features, highlighting its potential as a sustainable bio-memristive material for neuromorphic systems. Furthermore, the device exhibits sensitivity to the time interval between paired input pulses, simulating the neural response to interaural time differences (ITDs) in the auditory system. Although not a conventional acoustic sensor, its Δt-responsiveness based on synaptic behavior reveals promising potential in neuromorphic auditory perception and perceptual computing applications. This study provides a foundational synaptic unit for future artificial hearing systems capable of spatial sound localization.

## 1. Introduction

Hearing is one of the most important senses in the human body and plays an irreplaceable role in information acquisition and environmental adaptation [1]. In the human brain, the auditory system is an intricate, sophisticated, and efficient network [2]. Through our ears, sound signals are first captured and transmitted to the middle ear, and through the tympanic membrane, the auditory ossicles, sound is converted into nerve signals [3]. These nerve signals are then converted into electrical signals by auditory cells in the cochlea [4], which are then transmitted by the auditory nerve to the auditory cortex of the brain [5]. Neurons and synapses play a key role in this process, transmitting sound information to the brain for further processing [6]. Hearing is not only the simple perception of sound, but also involves the direction, distance, and speed of the sound source [7]. The accurate processing of this information allows us to sense where the sound is coming from and to react quickly, such as determining the direction of the sound source or determining the urgency of the sound [8]. As one of the core functions of auditory cognition, sound localization depends on the precise decoding of temporal cues, particularly the interaural time difference (ITD), which is processed by neural circuits in the brainstem. At present, the implementation of the sound localization function is based on CMOS circuits, and the principle is to detect the binaural sound signal receiving time difference and then realize the sound location function [9,10,11]. Duy Nguyen et al. used a field-programmable gate array to achieve real-time sound localization with two microphones and successfully located the sound source in a noisy environment with a low signal-to-noise ratio of 10 dB [12]. Jungdong Jin et al. implemented real-time sound localization based on a 0.13 µm CMOS processor. The arrival delay method is used to obtain the direction of the sound signal, but the design of the chip has higher requirements for the experimental environment [13]. Despite the effectiveness of these conventional systems, they are limited by high power consumption, hardware complexity, and latency caused by the separation between memory and processing units. With the explosive growth of real-time sound information data and the separation of processing units and memory [14], considerable time and power consumption are wasted in the process of data transmission, which cannot meet the growing needs of people [15].

As a new type of electronic component, a memristor has the characteristics that other components cannot match, that is, storage and computing integration [16], has great potential in data storage and computing performance [17], and can greatly reduce the power consumption and data transmission time between the processing unit and the storage unit. In terms of storage, memristors offer advantages such as good CMOS compatibility, low power consumption, and ultrahigh durability, making them ideal candidates for the next generation of high-density storage [18,19,20,21,22]. More importantly, the physical properties of memristors enable local data processing, making them suitable for distributed, brain-like computing architectures. In terms of computation, inspired by brain function, memristors can be regulated by different incentives to generate a variety of conductibles to simulate the activity of biological synapses and neurons, achieving unique neuromorphic computation [23,24,25,26,27,28,29,30,31,32,33,34]. Compared with traditional computing methods, this computing mode has the characteristics of the parallel processing of massive amounts of information [25,26,27].

To date, researchers have used a variety of materials and adopted a variety of structures to prepare high-performance memristors [28,29,30]. Compared with oxides [31,32], nitrides [33], and other organic materials [34,35,36,37,38], biomaterials have the advantages of simple processing [39], nontoxicity and harmlessness [40], biocompatibility [41], flexibility, and degradability. As sustainable and environmentally friendly materials, biomaterials offer unique compatibility with future wearable, implantable, and degradable neuromorphic systems. Many memristors prepared from biomaterials have been applied to the study of synaptic plasticity [42,43]. The device structure, electrical characteristics, and synaptic behavior of some of the memristors that have been published so far are shown in Table 1. The Ag/SNFs/ITO device prepared with silk nanofiber (SNFs) material as the medium layer has the electrical characteristics of a switching current ratio of 10^2^ and up to 180 cycles, and the device can be applied to memory imaging and logical operation functions [44]. The Au/silk:AgNO_3_/Ag device was prepared with silk fibroin and AgNO_3_ as the medium layer, the switching current ratio of the device was 3 × 10^6^, and the holding time was 10^3^ s under 60% humidity. These devices in the literature exhibit two neural behaviors: short-term plasticity (STP) and paired-pulse facilitation [45]. For the Ag/sericin/W device prepared with sericin as the dielectric layer, the biological memristor has a low set voltage and a cycle uniformity of 400 cycles. The device exhibits two neural behaviors: spiking rate-dependent plasticity (SRDP) and spiking time-dependent plasticity (STDP) [46]. The Al/CQD−chitosan/Au device is fabricated by using carbon quantum dots (CQDs) and a chitosan composite as the dielectric layer. The device has a high switching ratio of 10^6^ and repeatable and reliable bipolar resistance switching behavior. This study suggests that memory resistors based on bio-inspired nanocomposites can achieve biocompatibility, transparency, and green non-volatile storage devices (ReRAM) [47]. The Al/NaCas/ITO bio-memristor was prepared by using the natural casein (NaCas) extracted from milk as a raw material to synthesize water-soluble sodium caseinate as a medium layer. The device has a long retention time and good cycle durability [48]. Ag/AgNPs-CNCs/FTO devices prepared with a composite material of silver nanoparticles and cellulose nanocrystals (CNCs) as the medium layer exhibit both digital and analog switching characteristics and can simulate the synaptic behavior of organisms. The device has multiple neural behaviors including PPF, SRDP, an excitatory postsynaptic current (EPSC), long-term potentiation (LTP), long-term depression (LTD), paired-pulse depression (PPD), and post-tetanic potentiation (PTP) [49]. According to the data in Table 1, it can be seen that although the retention time of our device is 10^4^ s, slightly lower than the other two bio-memristor devices, Ag/SNFs/ITO and Al/NaCas/ITO (10^5^ s), the switching current ratios of Ag/SNFs/ITO and Al/NaCas/ITO are 10^2^ and 20, respectively, which are much lower than the switching current ratio of our proposed device (10^4^). Considering the switching current ratio, retention time, durability, and other electrical characteristics, the proposed Ag/SA:Au NPs/ITO device is competitive in terms of its performance. Moreover, in terms of applications, the device proposed in this study exhibits significant advantages in simulating neural synaptic behavior.

While previous bio-memristive devices have focused on mimicking short-term and long-term synaptic plasticity, few studies have addressed their potential in replicating higher-level auditory neural computations. Recently, bio-inspired systems based on cochlear architectures, such as the CAR-FAC model, have demonstrated the capability of localizing sound sources through interaural time difference (ITD) analysis [50,51]. In addition, CMOS neural circuits for ITD computation have been developed to emulate auditory localization in silicon-based platforms, further validating the computational significance of Δt-sensitive synaptic behavior [52]. These systems, however, often rely on sophisticated acoustic front-end components. In contrast, our proposed SA:Au NPs-based bio-memristor exhibits time-difference responsiveness via purely electrical pulse inputs, enabling the direct emulation of ITD neural coding at the circuit level. This positions the device as a viable building block for neuromorphic auditory localization without the need for conventional acoustic transducers.

In this paper, a resistive random-access memory (RRAM) device based on a composite of Scindapsus aureus (SA) and gold nanoparticles (Au NPs) is presented. The device demonstrates robust bipolar switching characteristics attributed to the formation and rupture of Ag conductive filaments. Its synaptic behaviors, including paired-pulse facilitation (PPF), spike-rate-dependent plasticity (SRDP), and spike-timing-dependent plasticity (STDP), are validated. Importantly, the device exhibits Δt-sensitive current responses, enabling a simulation of interaural time difference (ITD)-based direction selectivity, thereby contributing toward the development of neuromorphic auditory systems.

## 2. Experimental Section

Preparation of the Au NP solution: 0.1 g of chloroauric acid (Tianjin Baima Technology Co., LTD, Tianjin, China) was added to 1 L of deionized water, heated to boiling, stirred at 600 rpm/min, and 1 mL of a 1% aqueous sodium citrate solution was added. After the solution was boiled, it was heated continuously for 20 min and then cooled to room temperature to complete the preparation of the Au NP solution.

Fabrication of the Ag/SA:Au NPs/ITO/glass device: Firstly, Ag is widely used in resistive switching devices due to its excellent electrical conductivity and ability to form conductive filaments, which effectively facilitate current transport and provide high conductivity stability. Secondly, the SA:Au NP composite material, which combines the benefits of a natural organic material, contains polar functional groups that serve as charge trapping centers, improving the device’s retention and long-term stability. The doping of Au NPs enhances the electron-trapping capability and helps regulate the transition between high-resistance and low-resistance states. Finally, ITO serves as a transparent conductive electrode, providing good electrical contact and high transparency, which is advantageous for optoelectronic integration.

Fresh Scindapsus aureus leaves were first rinsed with distilled water to remove surface contaminants. The cleaned leaves were then blended in distilled water using a high-speed homogenizer to form a uniform pulp. The mixture was allowed to settle, and the supernatant was collected and filtered under vacuum through standard laboratory filter paper to obtain the SA extract solution, which was subsequently used as the dielectric precursor in the device fabrication process. The glass with the ITO electrode was cleaned by an ultrasonic instrument with alcohol, acetone, and deionized water for 15 min and then dried to remove the water on the surface of the ITO electrode. A total of 7.5 mL of the prepared Au NP solution was added to 0.5 mL of the prepared SA solution, and the doped solution was obtained by ultrasonication for 5 min. The SA:AuNP solution was drop-coated on the ITO/glass substrate by spin-coating at a speed of 500 rpm for 5 s and then at a high speed of 2000 rpm for 20 s. The formed SA:AuNP film was dried at 80 °C for 10 min. Using the masking plate, the active layer was deposited on the active layer using thermal evaporation (vacuum degree of 2 × 10^−3^ Pa) to precipitate 5 × 5 arrays of Ag electrodes on the active layer, and finally, annealed at 80 °C for 10 min to complete the preparation of Ag/SA:Au NP/ITO/glass resistive memories.

The experimental instruments used were an ultraviolet–visible spectrophotometer (UV/VIS, TU-1901) (Beijing Purkinje General Instrument Co., Ltd, Beijing, China), vacuum coating machine, drying box, ultrasonic cleaning machine, high-precision weighing balance, homogenizer, scanning electron microscope (Hitachi S-3400N) (Hitachi, Tokyo, Japan), semiconductor parameter tester (Keithley 4200) (Keithley, Solon, OH, USA), and transmission electron microscope (JEM-2100) (JEOL Co., Ltd., Tokyo, Japan). To ensure the repeatability and environmental robustness of the test results, all device measurements were conducted under ambient conditions at room temperature (22 ± 2 °C) and relative humidity of (45 ± 5%). The laboratory temperature and humidity control system was kept stable before and during all experiments to minimize the impact of environmental fluctuations on electrical performance. Additionally, throughout repeated cycling tests, the devices were maintained on a stable test platform with constant temperature and minimal mechanical disturbance to enhance the consistency and reliability of the measured performance.

## 3. Results and Discussion

**Figure 1:**
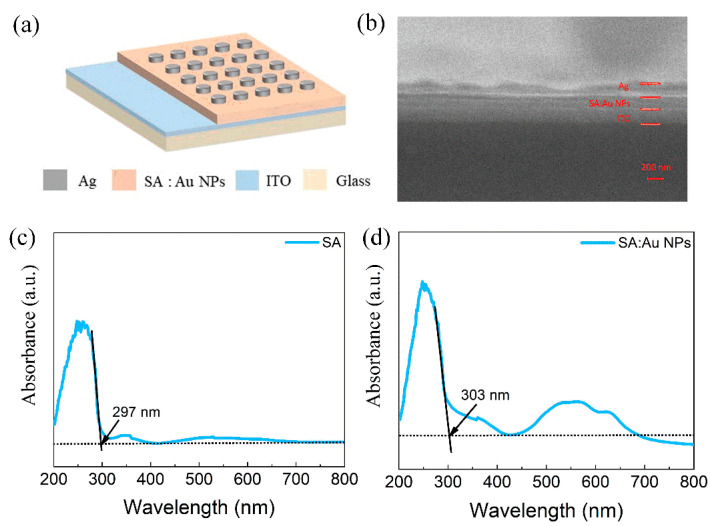
Ag/SA:Au NPs/ITO/glass device: (**a**) Schematic diagram of multi-layer structure of the device. (**b**) SEM images showing the thickness of each layer of the device. UV–visible absorption spectra of the dielectric films: (**c**) Pure SA film with absorption edge at 297 nm. (**d**) SA:Au NP composite film with red-shifted absorption edge at 303 nm, indicating bandgap narrowing induced by nanoparticle incorporation.

The structure diagram of the device is shown in Figure 1a. The device is composed of a Ag top electrode, SA:Au NP dielectric layer, ITO bottom electrode, and glass substrate. The cross-section of the Ag/SA:Au NPs/ITO device was observed by scanning electron microscopy, as shown in Figure 1b. From top to bottom are the Ag film (41 nm), SA:Au NP film (125 nm), ITO electrode (200 nm), and glass. Figure 1c,d show the UV–visible absorption spectra of the SA and SA:Au NPs, respectively. The absorption edge of the SA film is located at 297 nm, while that of the SA:Au NP film is red-shifted to 303 nm. According to the formula Eg = hc/λ, the corresponding bandgap widths are calculated to be 3.95 eV and 3.61 eV. This red shift indicates that the introduction of Au nanoparticles modifies the electronic structure of the dielectric film, possibly by introducing additional trap states or altering local charge distributions. The addition of Au NPs to the SA film significantly altered the bandgap width, demonstrating a redshift in the absorption spectrum. To further highlight the advantages of SA over other common natural materials in terms of bandgap characteristics, a comparison with three commonly used natural materials was made. The reported bandgaps of Egg Albumen and Tussah Hemolymph films are 3.67 eV and 2.61 eV, respectively [53,54]. In contrast, the SA film exhibits a higher bandgap of 3.95 eV, which contributes to enhanced device stability and resistance to interference. After doping with Au NPs, the bandgap decreased to 3.61 eV, yet this value is still higher than some common natural membrane materials. This structure, while maintaining good stability, benefits from the electron-trapping properties of Au NPs, enhancing the charge modulation and thereby improving the switching current ratio of the device.

**Figure 2:**
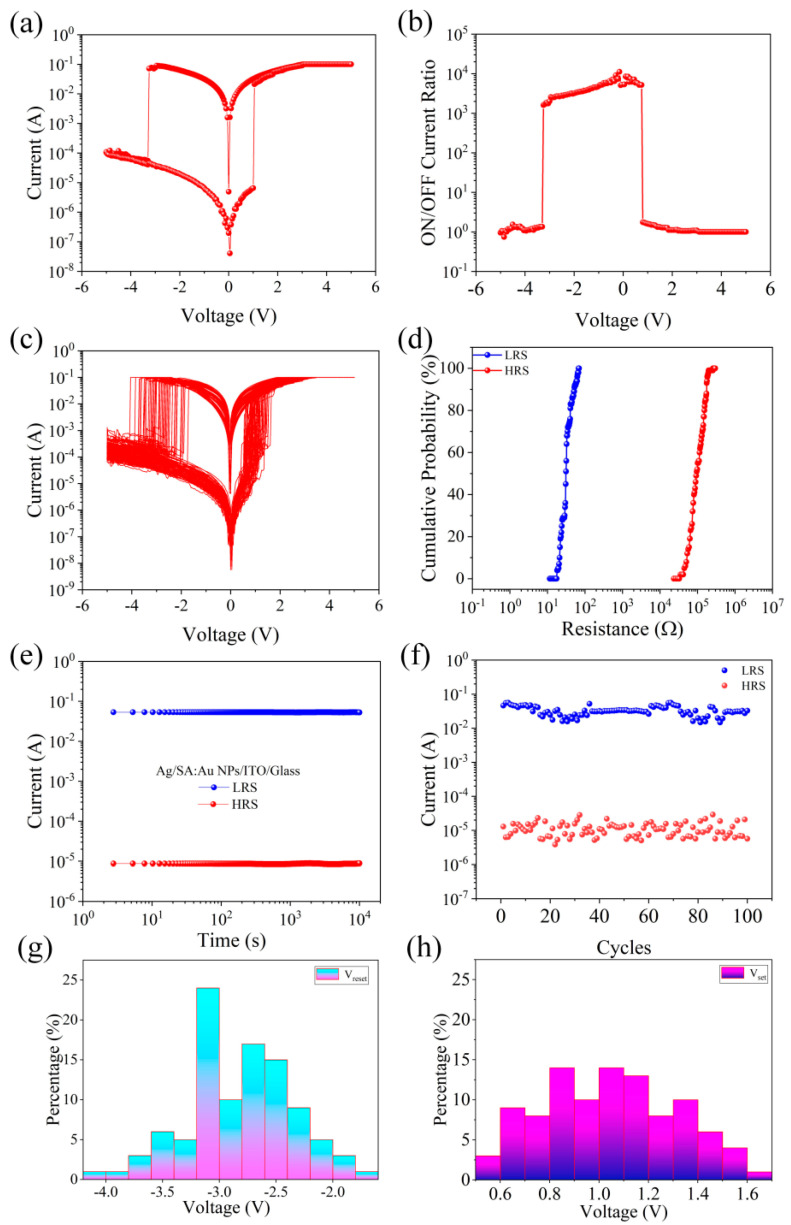
Electrical characteristics of the Ag/SA:Au NPs/ITO/glass device: (**a**) Typical I-V characteristic curve of the device. (**b**) The switching current ratio of the device. (**c**) I-V characteristic curve of the device with 100 cycles. (**d**) The cumulative probability curve of device resistance. (**e**) Current retention characteristics of devices. (**f**) Durability curve of the device. (**g**,**h**) Chart of threshold voltage distribution for devices.

The electrical characteristics of Ag/SA:Au NPs/ITO/glass devices are analyzed, and the I-V characteristics of the devices are tested at 0.1 A of limiting current (I_CC_) to prevent the life of the devices from being reduced due to too much current. Figure 2a shows the typical I-V characteristics of Ag/SA:Au NPs/ITO/glass, with the device exhibiting bipolar resistance switching characteristics. Figure 2b shows the switching current ratio of the Ag/SA:Au NPs/ITO/glass device. Figure 2b shows that the maximum switching current ratio of the device is approximately 10^4^. To study the repeatability of the Ag/SA:Au NPs/ITO/glass device, a unit of the top electrode array of the Ag/SA:Au NPs/ITO/glass device was selected, and continuous voltage scanning was repeatedly applied 100 times. The test results of the I-V curve are shown in Figure 2c. The stability of the device is good during continuous switching. Figure 2d shows the cumulative resistance probability of the Ag/SA:Au NPs/ITO/glass device at a voltage of 1 V, and the results show that the resistance distribution of the device is relatively concentrated. The retention performance of Ag/SA:Au NPs/ITO/glass devices under different resistance states at room temperature was tested. As shown in Figure 2e, the high-resistance state (HRS) and low-resistance state (LRS) of the devices were tested with time at a reading voltage of 1 V. The high- and low-resistance states were well maintained without significant attenuation, indicating that the devices have a long-term data retention ability. Figure 2f shows how the current values of the HRS and LRS change with the number of cycles at a reading voltage of 1 V for the devices. The low resistance is approximately 30 Ω, and the high resistance is distributed between 2 × 10^5^ Ω and 9 × 10^6^ Ω. The resistance value of Ag/SA:Au NPs/ITO/glass devices does not change much in 100 cycles. The reliability of the memristor is analyzed, and the threshold voltage distribution diagram is shown in Figure 2g,h. The threshold voltage distribution of the device is relatively concentrated and has high stability. To further validate the effect of Au nanoparticle doping, a reference device (Ag/SA/ITO/glass) was fabricated without Au NPs and tested under the same conditions. Its electrical performance, including the I–V characteristics, switching current ratio, retention, endurance, and threshold voltage distribution, is presented in Supporting Information Figure S1. These results show that the reference device exhibits a lower switching current ratio, broader resistance distribution, and reduced retention and endurance, confirming that the introduction of Au NPs enhances the electrical performance of the device.

**Figure 3:**
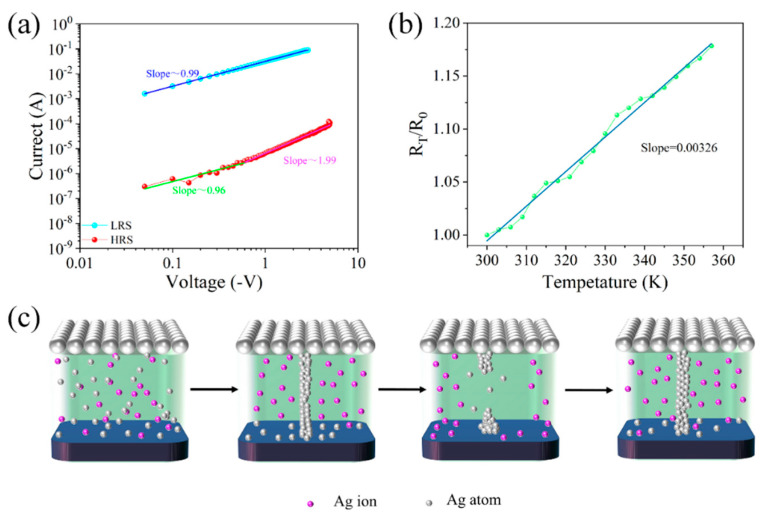
Ag/SA:Au NPs/ITO/glass device conductive mechanism: (**a**) I–V characteristic curve on the double logarithm axis. (**b**) Resistance with temperature test results. (**c**) Schematic diagram of the conductive mechanism of the device (silver conductive filament).

The conduction mechanism inside the Ag/SA:Au NPs/ITO/glass device was explored, and the I-V characteristic curve of the device was redrawn in log-double coordinates, as shown in Figure 3a. When the Ag/SA:Au NPs/ITO/glass device is in the LRS, the fitting slope of the device is approximately 0.99, indicating that ohmic conduction is the main conduction mechanism at this time. When the device is in the HRS, the slopes of the three parts of the log curve of the device are 0.96 and 1.99, which accords with the space charge-limited current (SCLC) theory. The relationship between the resistance value of the device and the temperature is further tested, as shown in Figure 3b, where R_0_ is the resistance value at 300 K, and R_T_ represents the resistance value at T. The fitted slope in the figure is approximately 3.26 × 10^−3^, and the conduction mechanism of the Al/SA:Au NPs/ITO/glass devices is combined. It is speculated that the resistance switching is mainly caused by the electrolysis and migration of Ag ions in the dielectric layer.

The conductive mechanism model of the device is shown in Figure 3c. When no bias voltage is applied, the particles in the dielectric layer are randomly distributed, and the device is in the HRS state. When a positive bias is applied to the Ag electrode, under the action of an external electric field, the Ag atoms in the device will be oxidized into Ag ions and migrate to the lower electrode of the dielectric layer, trapping electrons and reducing them to atoms, resulting in atomic deposition. When silver atoms accumulate to a certain extent, conductive filaments connecting the top electrode and the bottom electrode are formed in the dielectric layer, the electrical conductivity of the device is greatly improved, and the device becomes an LRS. However, the current is mainly transmitted through small local conductive wires, so the current flowing through these conductive wires generates a large amount of Joule heat, which causes the temperature of the filament to rise dramatically, eventually causing the filament to fuse and the entire device to switch back to the HRS. Finally, when a positive bias is applied to the top electrode, the device becomes low-resistance again under the action of an electric field.

In addition, SA contains abundant polar functional groups such as hydroxyl and carboxyl, which can act as transient trap centers under an electric field. These traps capture charge carriers and modulate the breaking behavior of conductive filaments, particularly in the HRS. The doped Au NPs provide uniformly distributed electron trapping sites due to their stable energy levels and high surface area, which further restrict free carrier transport and improve the retention and stability of the device. The double-logarithmic I–V curve characteristics suggest that the device follows a trap-controlled SCLC mechanism in the HRS, where the combined contribution of SA and Au NPs governs the conduction behavior. This interpretation aligns with recent findings on charge trapping-assisted switching in organic and nanoparticle-based memristors [55,56].

**Figure 4:**
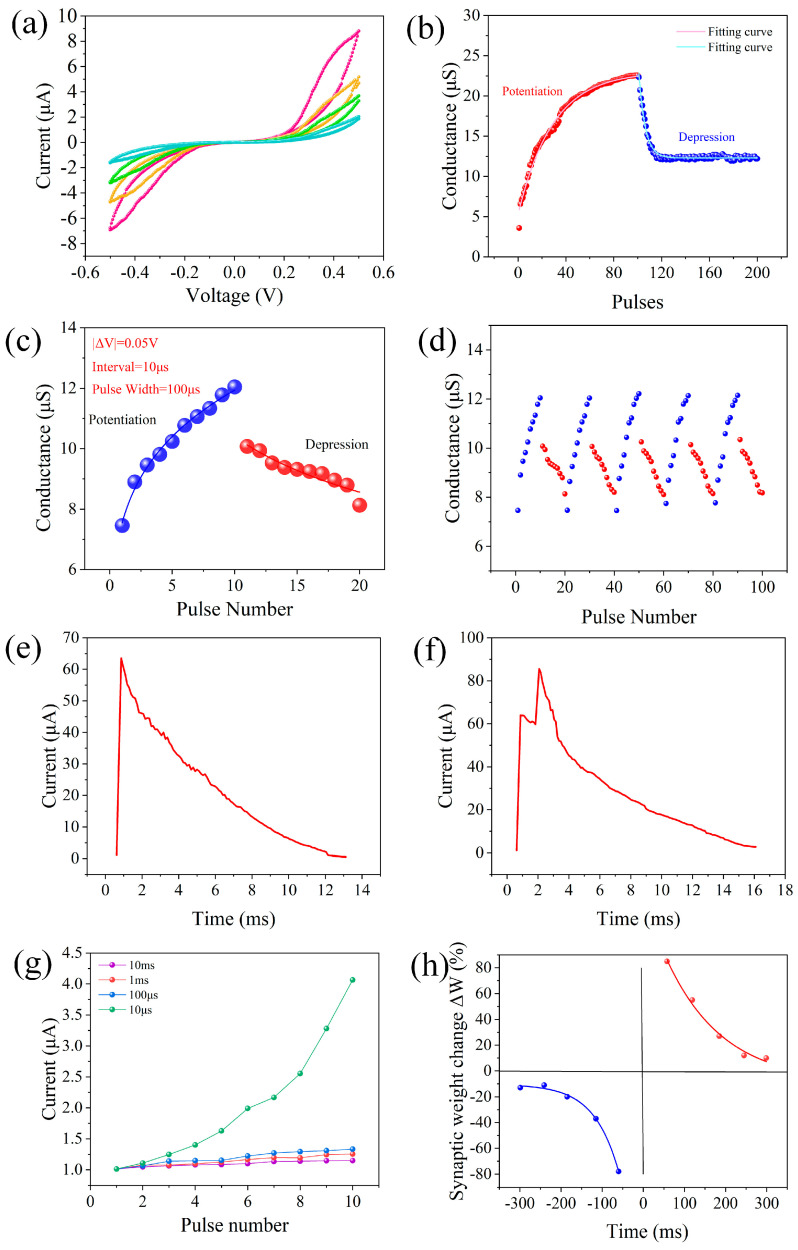
Ag/SA:Au NPs/ITO/glass device neurobehavior: (**a**) Conductivity modulation under repeated 0.5 V pulses (100 ms duration, 0.1 V read voltage after each pulse). (**b**) Long-term plasticity. (**c**,**d**) Conductance response under single-pulse stimulation and multipulse stimulation. (**e**) EPSC behavior. (**f**) PPF behavior under paired 1.5 V voltage spikes (1 ms pulse width, 10 μs interval). (**g**) SRDP behavior under pulse frequencies ranging from 100 Hz to 100 kHz (pulse width = 1 ms). (**h**) STDP behavior with Δt ranging from –100 ms to +100 ms (step = 10 ms, averaged over 5 repetitions).

In biological synapses, the strength of the connections between neurons, known as synaptic weights, can be regulated by successive pulses, which are known as the transmission properties of synapses. To study the synaptic transmission characteristics of the memristor, the current value of the device under four consecutive positive and negative voltages was measured, and the test results are shown in Figure 4a. When four positive voltages (0~0.5 V) are continuously applied to the top electrode of the memristor, the current of the memristor gradually decreases. Four consecutive 0.5 V voltage pulses were applied to the top electrode, each with a duration of 100 ms. The current was recorded after each pulse under a read voltage of 0.1 V. When four negative voltages (0~−0.5 V) are continuously applied, the magnitude of the negative current of the device increases. The results clearly show that applying repeated bias can change the conductance of the memristor. Since the conductance of the memristor is seen as a synaptic weight, these gradual changes in conductance mimic the transmission behavior in biological synapses.

One hundred consecutive forward pulses (+2 V, 1 ms) are applied to the device, and the change in the device conductance over time is recorded at a reading voltage of 0.1 V. The results are shown in Figure 4b, indicating that the conductance of the memristor increases with the increase in the number of forward pulses. After the forward pulses are removed, the conductance value of the device gradually decreases, and the final state remains at an equilibrium value. In Figure 4c, the Ag/SA:Au NPs/ITO/glass device is successively applied with a gradual increase in amplitude from −0.2 V to −0.65 V and a gradual decrease in amplitude from 1.2 V to 0.75 V (step size is 0.05 V), so that the conductivity of the device can be controllably increased or decreased. To simulate the “enhancement” and “suppression” behavior of synapses, where the pulse interval is 100 µs and the pulse width is 10 µs, the results show that Ag/SA:Au NPs/ITO/glass devices have great potential for realizing artificial electronic synapses. The results of applying a repeated pulse sequence to the device 10 times are shown in Figure 4d. The results show that the device has good response and stability after pulse application, and it has the possibility to simulate the synaptic plasticity of the human brain.

Figure 4e shows the current response of the memristor under a single spike (+1 V, 50 ms). The single spike triggers a sudden increase in the current, which subsequently decays to the initial state within 13 ms, which is attributed to the migration and diffusion of Ag ions, a process that is highly similar to the influx and efflux of Ca^2+^ in biological synaptic cells, mimicking EPSC behavior. This current response, characterized by a rapid rise and decay on the millisecond timescale, closely resembles the transient influx and subsequent diffusion of Ca^2+^ ions in biological synapses, indicating an excellent biomimetic performance in simulating synaptic transmission processes. When pairs of spikes are applied, a second spike can further enhance the response, showing a PPF of synaptic behavior, before the current caused by the first spike is fully attenuated. In this test, two pulses of a 1.5 V amplitude and 1 ms width were applied with an inter-pulse interval (∆t) of 10 μs. The reason for this result is that the superposition of the two spikes more effectively inhibits ion diffusion and allows silver ions to aggregate more efficiently, resulting in a larger change in their current, as shown in Figure 4f. This phenomenon can be attributed to the short interval between pulses, where the Ag ion diffusion induced by the first spike is not yet fully dissipated, allowing the second pulse to promote the further local aggregation of ions and resulting in an enhanced synaptic response.

Spiking rate-dependent plasticity (SRDP) in the biological nervous system reflects the relationship between the excitation frequency and synaptic weight. In a certain range, synaptic plasticity was positively correlated with the excitation frequency. For a memristor, the amount of change in its current depends largely on the frequency of the excitation applied to its ends. The SRDP behavior was tested using a series of voltage pulses at different frequencies (100 Hz to 100 kHz), with each pulse having a width of 1 ms. As shown in Figure 4g, the synaptic excitation of four frequencies is shown, and the influence of the excitation of different frequencies on the device current is different. When the excitation interval applied to the memristor is 10 ms, the device current changes very little. When the excitation interval is 10 μs, the device current changes greatly. These results indicate that the device exhibits good potential for emulating synaptic plasticity. In addition, we acknowledge that despite the excellent SRDP characteristics demonstrated by our device, several challenges remain for its large-scale application in future neuromorphic systems. For instance, device-to-device uniformity in large arrays, the further enhancement of the response speed, and long-term operational stability may all affect system-level scalability. In future work, we plan to optimize the device performance through material engineering and structural design to meet the higher requirements of neuromorphic computing systems in terms of reliability and energy efficiency.

As one of the important characteristics of synaptic plasticity, spiking time-dependent plasticity (STDP) is defined as the relative change in synaptic weight (Δ*W*) caused by the excitation of the anterior and posterior synaptic membranes. The relative timing ∆t between pre- and post-synaptic pulses was varied from –100 ms to +100 ms in steps of 10 ms, and each data point was averaged over five repetitions. The test results are shown in Figure 4h, where the synaptic weight changes as

(1) $$\Delta W=(G-G_{0})/G_{0}\times 100\%$$

where $G_{0}$ is the initial conductance value of the device and G is the conductance value of the device after pulse stimulation. It can be seen from the figure that the relative change in synaptic weight (Δ*W*) decreases with the increasing |Δ*t*|. STDP behavior can generally be fitted with the following formula

(2) $$\Delta W=A_{+}\exp(-\frac{\left|\Delta t\right|}{\tau_{+}})+\Delta W_{0+},\Delta t>0$$

(3) $$\Delta W=A_{-}\exp(-\frac{\left|\Delta t\right|}{\tau_{-}})+\Delta W_{0-},\Delta t<0$$

where $A_{+}$ and $A_{-}$ are scale factors, $\tau_{+}$ and $\tau_{-}$ are time constants, $\Delta W_{0+}$ is the value as we approach positive infinity, and $\Delta W_{0-}$ is the value as we approach negative infinity. In the above, $A_{+}$ = 145.19, $\tau_{+}$ = 137.48, $\Delta W_{0+}$ = 3.25, $A_{-}$ = −185.25, $\tau_{-}$ = −59.54, and $\Delta W_{0-}$ = −10.35.

**Figure 5:**
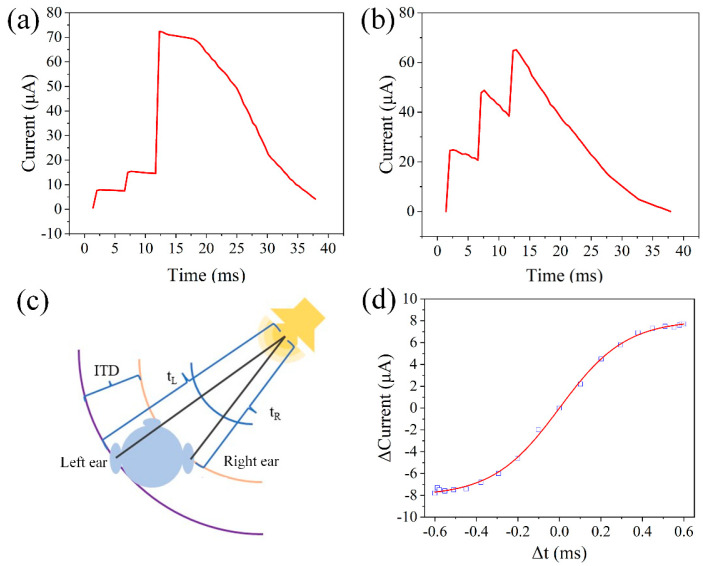
Spatiotemporal signal processing behavior of Ag/SA:Au NPs/ITO/glass device: (**a**) Current response under a pulse sequence of 1-2-3 (increasing spike arrival time), showing enhanced conductance and exceeding the threshold current, Ith. (**b**) Current response under a pulse sequence of 3-2-1 (decreasing spike arrival time), showing reduced conductance and sub-threshold output. (**c**) Conceptual schematic of interaural time difference (ITD)-based sound localization based on synaptic Δt-sensitivity. (**d**) Simulated current response as a function of Δt, derived from the synaptic behavior shown in Figure 4h.

Ag/SA:Au NPs/ITO/glass devices are used to simulate spatiotemporal pattern recognition. Suppose three pulses are numbered 1, 2, and 3, with synaptic weights decreasing sequentially from pulse 3 to pulse 1. Due to the memristor’s positive correlation between the spike arrival time and amplitude, later spikes produce stronger current responses. When pulses are applied in the 1-2-3 order (with an interval of 5 ms and amplitude of 1 V), the test results are shown in Figure 5a. The stronger response from later spikes allows the device to overcome the predefined threshold current, Ith (5 μA). Conversely, when pulses are applied in the reverse order (3-2-1), the results in Figure 5b show a lower response, failing to reach the threshold, due to the weaker synaptic weights of the later spikes. These results confirm the memristor’s capability to distinguish temporal sequences, enabling basic spatiotemporal recognition. Figure 5c presents a conceptual schematic for sound localization based on the device’s synaptic sensitivity to inter-spike timing. This model emulates the brain’s mechanism for determining the direction of a sound source by computing the interaural time difference (ITD = t_L_ − t_R_), where t_L_ and t_R_ represent the arrival times of sound at the left and right ears, respectively. In our system, two electrical pulses with a controlled time interval ∆t = t_L_ − t_R_ were applied to simulate auditory signals reaching each ear, and the corresponding device current responses were measured. Figure 5d shows the simulated output current under different ∆t conditions. The device exhibited a consistent current variation trend with an increasing ∆t, enabling the estimation of the sound direction based on the current magnitude. For example, when ∆t is 0.1 ms, the measured current change is approximately 2.2 μA, corresponding to a sound source located about 10° to the left of the right ear. It is important to note that Figure 5c is a conceptual diagram and Figure 5d is a simulated result based on the Δt-sensitive behavior demonstrated in Figure 4h. No actual acoustic front-end system or microphone array was implemented. The purpose of this model is to evaluate the potential of the device in neuromorphic auditory localization, rather than to establish a full sound perception system. These results further demonstrate the promise of the device in emulating brain-like functions based on synaptic conductance modulation.

## 4. Conclusions

In summary, Ag/SA:Au NPs/ITO/glass resistive memory is fabricated by using a composite material of green straw and gold nanoparticles as the dielectric layer. The device exhibits good bipolar resistance switching behavior. The device not only has a stable switching current ratio, but also has a low and stable threshold voltage. The device realizes synapse bionic behavior, such as conduction modulation, PPF behavior, and SRDP, and realizes sound source localization by sensing the time difference between the left and right ears to receive the input excitation. It provides a basic idea for realizing a brain-like bionic system integrating sense, memory, and computation.

## Figures and Tables

**Table 1:** Comparison of bio-memristor performance and synaptic behavior.

Device Structure	Switching Current Ratio	Retention Time (s)	Durability (times)	Threshold Voltage (V)	Synaptic Behavior	References
Ag/SA:Au NPs/ITO	10^4^	10^4^	100	V_SET_ = 1.02 V_RESET_ = −2.84	Enhancement and suppression EPSC, PPF, LTP, SRDP, STDP	This paper
Ag/SNFs/ITO	10^2^	10^5^	180	V_SET_ = 0.1~0.2 V_RESET_ = −0.2~−0.1	“AND” and “OR”	44
Au/silk:AgNO_3_/Ag	3 × 10^6^	10^3^	100	/	STP PPF	45
Ag/sericin/W	100	/	400	V_SET_ = 0.25	SRDP STDP	46
Al/CQD−chitosan/Au	10^6^	10^4^	160	V_SET_ = 0.75 V_RESET_ = −1	/	47
Al/NaCas/ITO	20	10^5^	180	/	/	48
Ag/AgNPs-TCNC/FTO	10^4^	10^4^	200	V_SET_ = 0.2 V_RESET_ = −0.2	LTP, LTD, EPSC, SRDP, PPF, PPD, PTP	49

## Data Availability

The data that support the findings of this study are available from the corresponding author upon reasonable request.

## Supplementary Materials

The following supporting information can be downloaded at: https://www.mdpi.com/article/10.3390/nano15090659/s1. Supporting Information is available and includes the electrical characterization of the reference device Ag/SA/ITO/glass, including I–V characteristics, the switching current ratio, retention, endurance, and threshold voltage distribution (Figure S1). These results support the role of Au NPs in improving the memristor performance.

## References

[1] Pang J., Peng S., Hou C., Wang X., Wang T., Cao Y., Zhou W., Sun D., Wang K., Rümmeli M. (2023). Applications of MXenes in human-like sensors and actuators. Nano Res..

[2] Di Liberto G.M., O’sullivan J.A., Lalor E.C. (2015). Low-frequency cortical entrainment to speech reflects phoneme-level processing. Curr. Biol..

[3] Kell A.J.E., Yamins D.L.K., Shook E.N., Norman-Haignere S.V., McDermott J.H. (2018). A task-optimized neural network replicates human auditory behavior, predicts brain responses, and reveals a cortical processing hierarchy. Neuron.

[4] Tichacek O., Mistrík P., Jungwirth P. (2023). From the outer ear to the nerve: A complete computer model of the peripheral auditory system. Hear. Res..

[5] Yun S.Y., Han J.K., Lee S.W., Yu J.M., Jeon S.B., Choi Y.K. (2023). Self-aware artificial auditory neuron with a triboelectric sensor for spike-based neuromorphic hardware. Nano Energy.

[6] Liu Y., Li E., Wang X., Chen Q., Zhou Y., Hu Y., Chen G., Chen H., Guo T. (2020). Self-powered artificial auditory pathway for intelligent neuromorphic computing and sound detection. Nano Energy.

[7] Escudero E.C., Peña F.P., Vicente R.P., Jimenez-Fernandez A., Moreno G.J., Morgado-Estevez A. (2018). Real-time neuro-inspired sound source localization and tracking architecture applied to a robotic platform. Neurocomputing.

[8] Grothe B., Pecka M., McAlpine D. (2010). Mechanisms of sound localization in mammals. Physiol. Rev..

[9] Wang Y., Mandal S. (2021). Bioinspired radio-frequency source localization based on cochlear cross-correlograms. Front. Neurosci..

[10] Wang Y., Mendis G.J., Wei-Kocsis J., Madanayake A., Mandal S. (2020). A 1.0–8.3 GHz cochlea-based real-time spectrum analyzer with Δ-Σ-modulated digital outputs. IEEE Trans. Circuits Syst. I Regul. Pap..

[11] Xu Y., Afshar S., Wang R., Thakur C.S., van Schaik A. (2021). A biologically inspired sound localization system using a silicon cochlea pair. Appl. Sci..

[12] Nguyen D., Aarabi P., Sheikholeslami A. Real-time sound localization using field-programmable gate arrays. Proceedings of the 2003 International Conference on Multimedia and Expo (ICME’03).

[13] Jungdong J. (2014). Real-time sound localization using generalized cross correlation based on 0.13 μm CMOS process. J. Semicond. Technol. Sci..

[14] Zhang W., Gao B., Tang J., Yao P., Yu S., Chang M.F., Yoo H.J., He Q., Wu H. (2020). Neuro-inspired computing chips. Nat. Electron..

[15] Xi Y., Gao B., Tang J., Chen A., Chang M.F., Hu X.S., Van Der Spiegel J., He Q., Wu H. (2020). In-Memory Learning with Analog Resistive Switching Memory: A Review and Perspective. Proc. IEEE.

[16] Gao S., Liu G., Chen Q., Xue W., Yang H., Shang J., Chen B., Zeng F., Song C., Pan F. (2018). Improving unipolar resistive switching uniformity with cone-shaped conducting filaments and its logic-in-memory application. ACS Appl. Mater. Interfaces.

[17] Wang L., Li J., Su W., Wen D. (2023). Nonvolatile Bioresistive Random Access Memory Based on *Glycine max* and Graphene Oxide. ACS Appl. Electron. Mater..

[18] Wang L., Zhu H., Zuo Z., Wen D. (2022). Full-function logic circuit based on egg albumen resistive memory. Appl. Phys. Lett..

[19] Wang L., Wei S., Xie J., Wen D. (2023). Bioartificial Synapses for Neuromorphic Computing. ACS Sustain. Chem. Eng..

[20] Wang L., Xie J., Wen D. (2023). Forming-free plant resistive random access memory based on the Coulomb blockade effect produced by gold nanoparticles. Phys. Chem. Chem. Phys..

[21] Kumar S., Davila N., Wang Z., Huang X., Strachan J.P., Vine D., Kilcoyne D.A.L., Nishibayashi Y., Williams R.S. (2017). Spatially uniform resistance switching of low current, high endurance titanium–niobium-oxide memristors. Nanoscale.

[22] Papadopoulos S., Agarwal T., Jain A., Taniguchi T., Watanabe K., Luisier M., Emboras A., Novotny L. (2022). Ion Migration in Monolayer Mo S 2 Memristors. Phys. Rev. Appl..

[23] Wang L., Li J., Su W., Wen D. (2023). Photoelectric biomemristors for artificial visual perception systems. Appl. Mater. Today.

[24] Wang L., Qu J., Li J., Wen D. (2023). Realization of artificial synapses using high-performance soybean resistive memory. J. Alloys Compd..

[25] Wang L., Qu J., Li J., Wen D. (2023). Full Hardware Image Encryption for Oscillating Memristor Circuits. Adv. Mater. Technol..

[26] Vidiš M., Plecenik T., Moško M., Tomašec S., Roch T., Satrapinskyy L., Grančič B., Plecenik A. (2019). Gasistor: A memristor based gas-triggered switch and gas sensor with memory. Appl. Phys. Lett..

[27] Luo Y., Zhao D., Zhao Y., Chiang F.K., Chen P., Guo M., Luo N., Jiang X., Miao P., Sun Y. (2015). Evolution of Ni nanofilaments and electromagnetic coupling in the resistive switching of NiO. Nanoscale.

[28] Chen J., Xu J., Chen J., Gao L., Yang C., Guo T., Zhao Y., Xiao Y., Wang J., Li Y. (2022). High-performance memristor based on MoS2 for reliable biological synapse emulation. Mater. Today Commun..

[29] Wang T.Y., Meng J.L., Li Q.X., Chen L., Zhu H., Sun Q.Q., Ding S.J., Zhang D.W. (2021). Forming-free flexible memristor with multilevel storage for neuromorphic computing by full PVD technique. J. Mater. Sci. Technol..

[30] Xu X., Zhou X., Wang T., Shi X., Liu Y., Zuo Y., Xu L., Wang M., Hu X., Yang X. (2020). Robust DNA-Bridged Memristor for Textile Chips. Angew. Chem. Int. Ed..

[31] Qi M., Cao S., Yang L., Qi Y., Shi L., Wu Z. (2020). Uniform multilevel switching of graphene oxide-based RRAM achieved by embedding with gold nanoparticles for image pattern recognition. Appl. Phys. Lett..

[32] Ahn M., Park Y., Lee S.H., Chae S., Lee J., Heron J.T., Kioupakis E., Lu W.D., Phillips J.D. (2021). Memristors Based on (Zr, Hf, Nb, Ta, Mo, W) High-Entropy Oxides. Adv. Electron. Mater..

[33] Lin C.Y., Chen J., Chen P.H., Chang T.C., Wu Y., Eshraghian J.K., Moon J., Yoo S., Wang Y.H., Chen W.C. (2020). Adaptive synaptic memory via lithium ion modulation in RRAM devices. Small.

[34] Yuan L., Liu S., Chen W., Fan F., Liu G. (2021). Organic memory and memristors: From mechanisms, materials to devices. Adv. Electron. Mater..

[35] Zhang M., Ma C., Du D., Xiang J., Yao S., Hu E., Liu S., Tong Y., Wong W.Y., Zhao Q. (2020). Donor–acceptor metallopolymers containing ferrocene for brain inspired memristive devices. Adv. Electron. Mater..

[36] Majumdar S., Tan H., Pande I., Van Dijken S. (2019). Crossover from synaptic to neuronal functionalities through carrier concentration control in Nb-doped SrTiO3-based organic ferroelectric tunnel junctions. APL Mater..

[37] Shen Z., Zhao C., Qi Y., Xu W., Liu Y., Mitrovic I.Z., Li Y., Zhao C. (2020). Advances of RRAM devices: Resistive switching mechanisms, materials and bionic synaptic application. Nanomaterials.

[38] Park H.L., Kim M.H., Kim H., Lee S.H. (2021). Self-Selective Organic Memristor by Engineered Conductive Nanofilament Diffusion for Realization of Practical Neuromorphic System. Adv. Electron. Mater..

[39] Mao J.Y., Zhou L., Ren Y., Yang J.Q., Chang C.L., Lin H.C., Chou H.H., Zhang S.R., Zhou Y., Han S.T. (2019). A bioinspired electronic synapse using solution processable organic small molecule. J. Mater. Chem. C.

[40] Huang W.Y., Chang Y.C., Sie Y.F., Yu C.R. (2021). Biocellulose substrate for fabricating fully biodegradable resistive random access devices. ACS Appl. Polym. Mater..

[41] Sun W.J., Zhao Y.Y., Zhou J., Cheng X.F., He J.H., Lu J.M. (2019). One-Step Fabrication of Bio-Compatible Coordination Complex Film on Diverse Substrates for Ternary Flexible Memory. Chem. A Eur. J..

[42] Satapathi S., Raj K., Afroz M.A. (2022). Halide-perovskite-based memristor devices and their application in neuromorphic computing. Phys. Rev. Appl..

[43] Kumar M., Ban D.K., Kim S.M., Kim J., Wong C.P. (2019). Vertically aligned WS2 layers for high-performing memristors and artificial synapses. Adv. Electron. Mater..

[44] Zhang Y., Fan S., Niu Q., Han F., Zhang Y. (2022). Intrinsically ionic conductive nanofibrils for ultrathin biomemristor with low operating voltage. Sci. China Mater..

[45] Zhao M., Wang S., Li D., Wang R., Li F., Wu M., Liang K., Ren H., Zheng X., Guo C. (2022). Silk protein based volatile threshold switching memristors for neuromorphic computing. Adv. Electron. Mater..

[46] Rong H., Zhang M., Liang X., Liu C., Saadi M., Chen X., Yao L., Zhang Y., He N., Hu E. (2023). Demonstration of electronic synapses using a sericin-based biomemristor. Appl. Phys. Express.

[47] Raeis-Hosseini N., Georgiadou D.G., Papavassiliou C. (2022). High on/off ratio carbon quantum dot–chitosan biomemristors with coplanar nanogap electrodes. ACS Appl. Electron. Mater..

[48] Saha M., Nawaz S.M., Keshari B.K., Mallik A.N. (2022). Natural-casein-based biomemristor with pinched current–voltage characteristics. ACS Appl. Bio Mater..

[49] Hussain T., Abbas H., Youn C., Lee H., Boynazarov T., Ku B., Jeon Y.R., Han H., Lee J.H., Choi C. (2022). Cellulose nanocrystal based Bio-Memristor as a green artificial synaptic device for neuromorphic computing applications. Adv. Mater. Technol..

[50] Han Y., Zhang Y., Chen X., Zeng W., Yang J., Zhao Y. (2021). Sound Source Localization Based on Interaural Time Difference Using a Biologically Inspired Cochlear Model. Appl. Sci..

[51] Wang W., Yin X., Zhao H., Liang Y. (2021). Cochlear-Inspired Neural Cross-Correlogram Architecture for Sound Localization. Front. Neurosci..

[52] Wang S., Song Y., Wang Y., Yang Y., Wei Y., Liu X., Hu X. (2020). A Biologically Inspired Cochlear Nucleus Neural Network for Sound Source Localization Using ITD Cues. IEEE Trans. Circuits Syst. I Regul. Pap..

[53] Wang L., Wen D. (2017). Nonvolatile Bio-Memristor Based on Silkworm Hemolymph Proteins. Sci. Rep..

[54] Wang L., Zhang Z., Liu Y., Yang H., Guo S., Liu Z., Hu X. (2021). The Role of Egg Albumin Membranes in the Electronic Properties of Resistive Switching Devices. Nanomaterials.

[55] Moon T., Soh K., Kim J.S., Yang J.J., Yoon J.H. (2024). Leveraging volatile memristors in neuromorphic computing: From materials to system implementation. Mater. Horiz..

[56] Shi C., Park Y., Bae Y., Lee S. (2024). Multiparametric AFM insights into electron transport mechanisms in biomemristors. Mater. Today Phys..

