# Supplementary material for: Scindapsus Aureus Resistive Random-Access Memory with Synaptic Plasticity and Sound Localization Function
Source: Nanomaterials (Basel). 2025 Apr 26;15(9):659. doi: 10.3390/nano15090659 (PMC12073795; doi:10.3390/nano15090659)
Supplement: Supplementary file 1 [file nanomaterials-15-00659-s001.zip › nanomaterials-3333575-supplementary.pdf]

Supporting Information

Scindapsus aureus resistive random access memory with synaptic plasticity and sound localization function

*Lu Wang *, Jiachu Xie, Wantao Su, Zhenjie Du and Mingzhu Zhang*

School of Electronic Engineering, Heilongjiang University, Harbin, 150080, China
Email: wanglu@hlju.edu.cn

S1. Electrical Characteristics of the Reference Device

To evaluate the baseline performance and isolate the effect of Au NPs doping, we fabricated a reference device using a pure SA-based dielectric layer (Ag/SA/ITO/glass), without any nanoparticle incorporation. The electrical characteristics are summarized in Fig. S1. Compared with the Au NPs-doped counterpart, the reference sample exhibited a lower switching current ratio, reduced endurance stability, and a broader distribution of threshold voltages. These results confirm that the introduction of Au NPs enhances the trap density within the dielectric, thereby improving carrier modulation and synaptic-like switching performance.

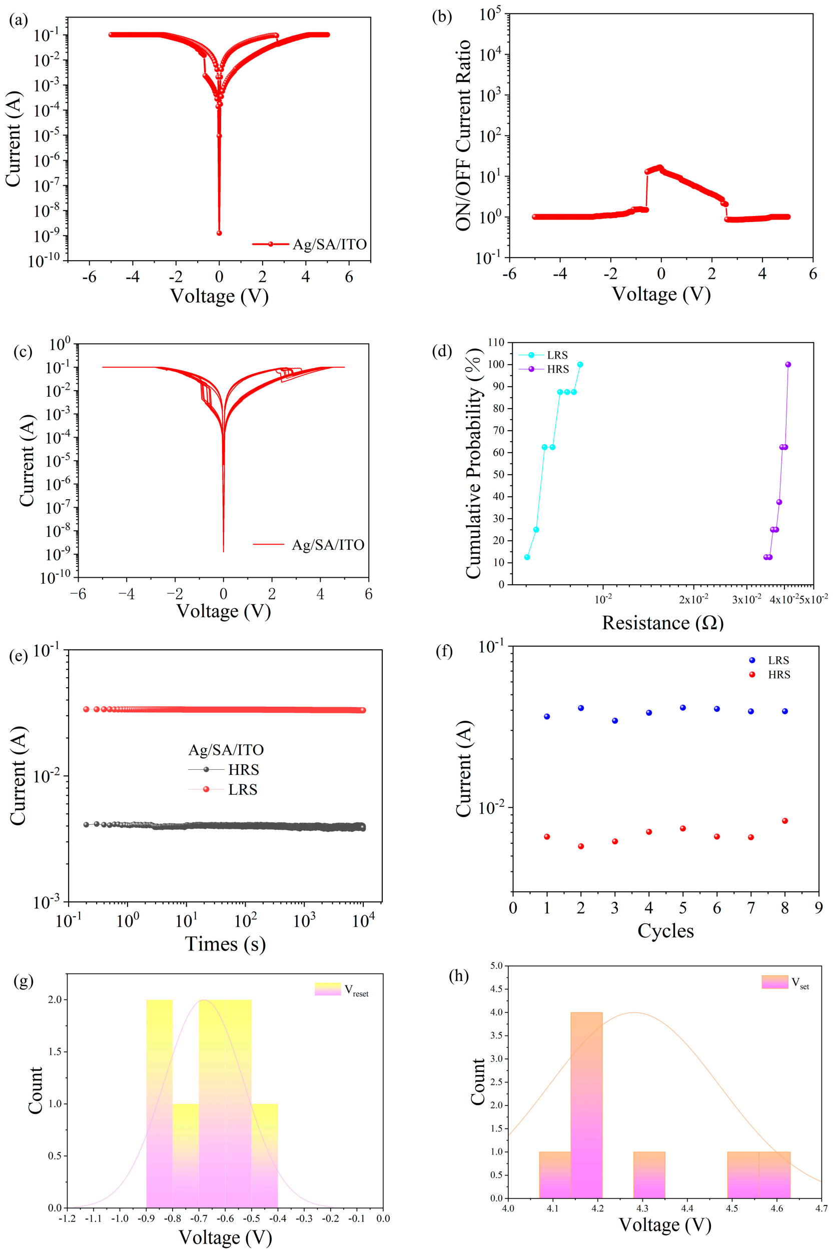

Figure S1. Electrical characterization of the Ag/SA/ITO/glass control device (without Au NPs):

(a) Typical I–V curve under bipolar voltage sweep.

(b) Switching current ratio.

(c) I–V characteristics over 8 consecutive cycles.

(d) Cumulative distribution of high- and low-resistance states.

(e) Retention performance measured over 10^4 s at 1 V read voltage.

(f) Endurance test under 8 switching cycles.

(g–h) Distribution of threshold voltages (set/reset) across multiple tests.
